# Selecting the Best Approach for the Treatment of Multiple Non-Metastatic Hepatocellular Carcinoma

**DOI:** 10.3390/cancers14235997

**Published:** 2022-12-05

**Authors:** Gianluca Cassese, Ho-Seong Han, Jai Young Cho, Hae-Won Lee, Boram Lee, Roberto Ivan Troisi

**Affiliations:** 1 Department of Surgery, Seoul National University College of Medicine, Seoul National University Bundang Hospital, 166 Gumi-ro, Bundang-gu, Seongnam-si 13620, Republic of Korea; 2 Department of Clinical Medicine and Surgery, Division of Minimally Invasive and Robotic HPB Surgery and Transplantation Service, Federico II University, 80138 Naples, Italy

**Keywords:** multiple hepatocellular carcinoma, multinodular hepatocellular carcinoma, laparoscopic liver resection, transarterial chemo-embolization, radiotherapy, thermal ablations

## Abstract

**Simple Summary:**

The best therapy for patients with multiple HCC within the Milan Criteria is liver transplantation (LT). However, LT cannot be offered to all the patients. For the intermediate staged multiple HCC trans-arterial chemoembolization (TACE) still remains the treatment of choice. However, a growing body of evidence is showing better outcomes after surgery than TACE. Trans-arterial radioembolization and stereotaxic body radiation therapy can also play an important role in this setting. Furthermore, the role of minimally invasive liver surgery (MILS) for patients with multiple HCC is still debated.

**Abstract:**

According to the Barcelona Clinic Liver Cancer (BCLC) staging system, the optimal strategy for patients with multiple HCC within the Milan Criteria is liver transplantation (LT). However, LT cannot be offered to all the patients due to organ shortages and long waiting lists, as well as because of the advanced disease carrying a high risk of poor outcomes. For early stages, liver resection (LR) or thermal ablation (TA) can be proposed, while trans-arterial chemoembolization (TACE) still remains the treatment of choice for intermediate stages (BCLC-B). Asian guidelines and the National Comprehensive Cancer Network suggest LR for resectable multinodular HCCs, even beyond Milan criteria. In this scenario, a growing body of evidence shows better outcomes after surgical resection when compared with TACE. Trans-arterial radioembolization (TARE) and stereotaxic body radiation therapy (SBRT) can also play an important role in this setting. Furthermore, the role of minimally invasive liver surgery (MILS) specifically for patients with multiple HCC is still not clear. This review aims to summarize current knowledge about the best therapeutical strategy for multiple HCC while focusing on the role of minimally invasive surgery and on the most attractive future perspectives.

## 1. Introduction

Hepatocellular carcinoma (HCC), with an estimated incidence of around 900,000 cases per year, accounts for the seventh most common cancer worldwide and the third leading cause of cancer-related death [1]. HCC prognosis is related to the stage of diagnosis, reaching 5-yr overall survival rates (OS) of 50–70% at early stages due to technical and technological advances as well as improvements in perioperative management [2]. However, recurrence still represents a major issue, with a rate of 70% after liver resection (LR) and 20% after liver transplantation (LT) [3].

Surgery represents the cornerstone treatment for HCC. LT is the best therapeutic option, aiming to treat both HCC and underlying chronic liver disease, including liver cirrhosis. Nonetheless, owing to the organ shortage, there is a long waiting time carrying a high risk of dropout for tumor progression [4]. Accordingly, both LR and thermal ablations (TA) are actually considered the first-line strategy for well-compensated HCC patients according to all Western guidelines. LT is essentially reserved for patients who are not candidates for LR due to impaired liver function or for patients with negative prognostic factors on specimen examination after a previous resection [5,6].

Among the different risk factors of HCC recurrence, an important role is played by the number of tumors. Indeed, the tumor number is an important parameter within all selection criteria for LT. Classically, the Milan criteria are the most widely used transplant criteria, and they restrict the applicability of LT to patients with fewer than three nodules, all smaller than 3 cm [7]. Similar restrictions are indicated by the University of California San Francisco criteria (UCSF) as well as the up-to-seven criteria that are even more rigid [8,9]. Thus, the therapeutic management of patients with multiple HCC who do not meet such criteria is still debated; the same applies to HCC patients who meet the criteria but with little possibility of receiving an organ in the sort time. According to the most recent European recommendations, LR is indicated for very early and early stages of the Barcelona Clinic Liver Cancer staging system (BCLC 0/A), while TA is recommended in cases of bi- or tri-focal tumors ≤3 cm, if LT is not feasible [10]. For more than three nodules, patients are staged as BCLC-B, and they are recommended to undergo trans-arterial chemoembolization (TACE). However, the latest guidelines from the National Comprehensive Cancer Network (NCCN) recommend LR also in cases of multiple HCCs [11]. Furthermore, Asian guidelines also suggest LR for multinodular HCC [12].

In this scenario, the laparoscopic approach has widely spread in liver surgery, becoming the standard of care in referral tertiary centers [13,14]. However, there are some challenging situations in which the role of laparoscopic liver resection (LLR) is still debated, and the resection of multiple HCC is definitely one of them [15].

This review aims to clarify the prognostic role of liver resection for multiple HCC, then focus on the actual and future roles of minimally invasive liver surgery (MILS) and other available strategies.

## 2. Current Evidence about the Management of Multiple HCC

### 2.1. Prognostic Role of Surgical Treatment for Multiple HCC

The role of LR in multiple HCC is still debated, especially in cases of an underlying severe liver disease. According to the last BCLC criteria, if the number of nodules is less than three, with all nodules smaller than 3 cm (within the Milan criteria), patients should undergo LT. If LT is not available, TA should be the first option [6]. If the number or the size of the lesions overwhelms the Milan criteria or the liver function is not well preserved, such patients are classified as intermediate stage according to BCLC, with western guidelines actually contraindicating surgery in the light of worsened outcomes [5,6,16]. BCLC-B patients are supposed to undergo TACE. However, many previous papers reported better outcomes after LR than TACE in patients with multiple HCC, even if they were not considered ideal candidates for LR (Table 1). Tumor number has been shown to be a risk factor for worse oncological outcomes [17,18,19,20]. In 2008, Ishizawa et al. reported a wide series of encouraging results after LR for multiple HCC, concluding that it should not be considered a contraindication to surgery [17]. Similarly, Ho et al. in 2009 showed interesting results from a study on 1065 patients with multiple HCC, with a median survival after surgical resection significantly better than TACE (37.9 months vs. 17.3 months, respectively, *p* < 0.001). The 1-yr, 3-yr, and 5-yr OS after LR were 77.4%, 51.9%, and 36.6%, and the advantage in terms of survival was still present after subgroup analysis according to the Milan criteria. Later, clear benefits of the surgical treatment came from a randomized controlled trial, where LR provided a better OS than TACE for patients beyond the Milan criteria (1-yr, 2-yr, and 3-yr OS of 76.1%, 63.5%, vs. 51.5% vs. 51.8%, 34.8%, and 18.1%, respectively, *p* < 0.001) [21].

Finally, a very recent meta-analysis of high-quality studies (including only one randomized controlled trial and six propensity-score matching (PSM) comparative studies) focused specifically on 2487 patients with multiple HCCs staged as BCLC-B (1245 in the LR group and 1242 in the TACE group) [22]. Patients undergoing LR had significantly higher OS than TACE group (HR, 1.65, *p* = 0.026) and 1-, 3-, and 5-year survival rates (Odds Ratio: 1.96, *p* = 0.0005; OR: 2.92, *p* = 0.0001; OR: 2.60, *p* < 0.00001, respectively).

The maximum number, location, and type of recurrence of tumors beyond which the risks overwhelm the benefits of liver resection are still not clear. Recent literature suggests that the prognostic role of tumor number is correlated with tumor size. The tumor burden score (TBS) is based on this concept and has shown the best discriminative ability on survival outcomes when compared to MC and other tumor-specific scores [23]. TBS, defined as the distance from the origin of a Cartesian plane having tumor size on its *x*-axis and tumor number on its *y*-axis, was initially proposed as a prognostic tool for colorectal liver metastases (CRLM) [24]. Tsilimigras et al. recently showed a strong correlation within TBS and survival outcomes in comparison to the BCLC criteria in a multicenter study on 1053 patients with HCC undergoing liver resection [25]. Patients with the same TBS had similar outcomes, irrespective of their BCLC stage: patients with a BCLC-B stage and a medium TBS had a higher 5-yr OS than BCLC-A stages with a high TBS (58.9% vs. 45%, *p* = 0.005). In the multivariable survival analysis adjusted for the competing risk factors, not only TBS but also alpha-fetoprotein (AFP) level was correlated with prognosis, as were pathologic parameters of tumor aggressiveness and underlying liver cirrhosis [25]. These findings suggest that tumor biology should be considered when dealing with HCC management, and in particular with LT, considering the importance of correct graft allocation due to organ shortage [26]. The Metroticket 2.0 is an example of integrated model based on tumor size, tumor number, and AFP level to determine the survival outcomes from HCC-related factors after liver transplantation, useful to refine selection criteria for LT for HCC. Recently, Kokudo et al. reported survival outcomes after LR for multiple HCCs from a large cohort of 1170 cases. The median OS was 9.74 years in the case of 1 tumor, 6.36 years for 2 lesions, 7.21 years for 3 lesions, 3.31 years in the case of 4 HCCs, and 3.48 years in the case of 5 lesions [27]. The difference in median OS was significantly lower for patients with more than 3 HCC nodules (*p* < 0.0001). Regarding the treatment-related outcomes, the patients who had undergone LR had longer survival after recurrence (SAR) when compared to other treatments (8.32 vs. 3.19 years; *p* < 0.001). Similar results came from a nationwide Japanese study on 2178 patients, comparing 1089 LR with 1089 TACE. The 5-yr OS was higher after LR (60.0% vs. 41.6%, *p* < 0.001), also for tumors larger than 30 mm (53.0% vs. 32.7%, *p* < 0.001). The multivariate analysis showed age, AFP level, bilirubin level, tumor size, vascular invasion, and previous TACE to be independent predictors of worse prognosis in the case of multinodular HCC. Indeed, current Asian guidelines recommend LR of multiple HCCs (up to three according to Japanese recommendations, regardless of the number according to the Korean’s), regardless of size [12,28,29].

**Figure 1:**
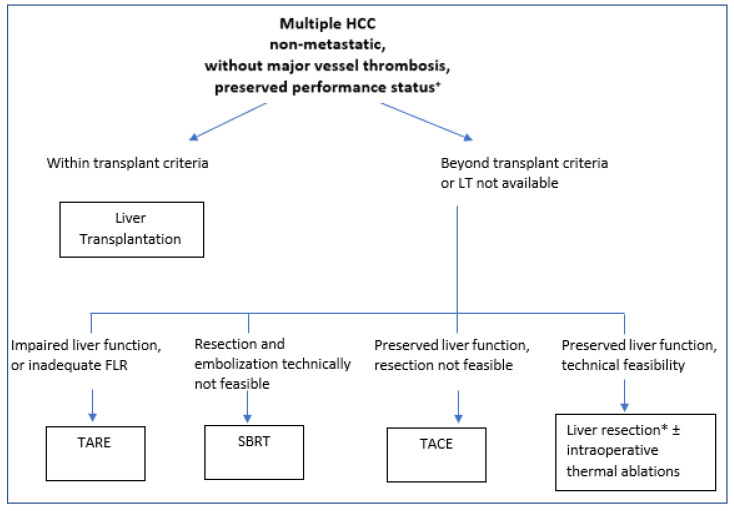
Proposed treatment algorithm for multiple non-metastatic HCC. ^+^ All patients need to be evaluated and discussed in a multidisciplinary team approach, and treatment must be tailored to every specific case. * Laparoscopic approaches should be preferred in centers with adequate expertise and in cirrhotic patients. FLR: future liver remnant; SBRT: stereotactic body radiation therapy; TARE: trans-arterial radio-embolization; TACE: trans-arterial chemo-embolization; HCC: hepatocellular carcinoma.

Based on these findings and our experience, we strongly believe that in selected patients with up to three HCC nodules, a good performance status and well compensated liver function, when LT is not available, surgery should be the first strategy. TACE should be reserved for sequential combined treatment or in cases of more advanced disease or that is technically unresectable. A therapeutic algorithm is proposed in Figure 1.

**Table 1:** Studies comparing Surgery and TACE in BCLC-B patients. OS: overall survival; TACE: trans-arterial chemoembolization; HCC: hepatocellular carcinoma; BCLC: Barcelona clinic liver cancer.

Year	Authors	Study Design	Compared Groups	Sample Size	Additional Inclusion Criteria	3-yr OS (%)	5-yr OS (%)	Median OS (Months)	*p*-Value
2010	Lin et al. [30]	Retrospective	Surgery vs. TACE	93 vs. 73	Intermediate stage	49 vs. 2			<0.001
2011	Luo et al. [31]	Prospective	Surgery vs. TACE	85 vs. 83	Intermediate stage and solitary tumor ≥5 cm	35.3 vs. 26.0	23.9 vs. 18.9		0.26
2014	Yin et al. [21]	Randomized controlled trial	Surgery vs. TACE	88 vs. 85	Outside the Milan criteria without MVI	51.5 vs. 18.1			<0.001
2014	Jianyong et al. [32]	Retrospective	Surgery vs. TACE	433 vs. 490	Intermediate stage	71.1	61.2		<0.001
2015	Ciria et al. [33]	Retrospective	Surgery vs. TACE	36 vs. 44	Intermediate stage	52.8 vs. 47.7	44.4 vs. 38.6		0.23
2016	Kim et al. [34]	Retrospective	Surgery vs. TACE	52 vs. 225	Intermediate stage	65.0 vs. 39.2	51.8 vs. 27.9		0.02
2016	Zhao et al. [35]	Retrospective	Surgery vs. TACE	274 vs. 169	Intermediate stage	46 vs. 15	37 vs. 12		<0.001
2017	Tada et al. [36]	Retrospective	Surgery vs. TACE	132 vs. 132	Intermediate stage	63.4 vs. 53	53.1 vs. 34.1		0.01
2019	Fukami et al. [37]	Nationwide retrospective propensity score—matched	Surgery vs. TACE	1089 vs. 1089	Multiple Child A HCCs within the Milan criteria	75 vs. 62.5	60 vs. 41.6		<0.001
2021	Lu et al. [38]	Retrospective propensity score—matched	Surgery vs. TACE	225 vs. 717	Intermediate stage			67.4 vs. 29.9	<0.0003

### 2.2. Role of Thermal Ablation

TA for HCC is accepted as a curative treatment option in many HCC treatment guidelines due to nature of the procedure being minimally invasiveness and the survival outcomes that have been reported to be comparable to LR for very-early stages and nodules <3 cm [6,39,40,41].

As previously discussed, many previous papers reported that LR could provide better survival outcomes than TACE in select patients with multiple HCC. However, in cirrhotic patients, post-hepatectomy liver failure (PHLF) due to an inadequate future remnant liver (FRL) is still a major issue [42]. Therefore, some patients are not suitable for multiple liver resections, especially patients with multifocal HCC beyond the Milan criteria. However, according to the BCLC algorithm, RFA is actually indicated only for early and very early stage patients who meet the Milan criteria [6]. Nevertheless, also in this setting, previous PSM studies showed that LR could provide better disease-free survival (DFS) than radiofrequency ablation (RFA) or TACE, with similar survival outcomes in cases of multinodular HCC within Milan criteria, even if the postoperative complication rate was higher after surgery [43].

Percutaneous TA has shown some important limits. First, a lesion located close to the diaphragm, in posterosuperior segments, or close to the biliary duct or major vessels is not treatable owing to the high risk of complications [44]. Secondly, preprocedural ultrasounds (US) can miss a lesion smaller than 3 cm in more than 20% of cases [45]. A prospective multicenter Korean study on 898 patients reported a rate of pre-procedural US detection of 74.7% [46]. In multivariate analysis, tumor size, distance between the tumor and the diaphragm, liver cirrhosis, and macronodular cirrhosis were statistically significant factors affecting US detection (each *p* < 0.05). To overcome these limitations, TA can be applied with a surgical approach. Previous meta-nalyses suggested better oncological outcomes for surgical TA, resulting in superior local control independent of tumor size (*p* < 0.0001) [47]. The authors concluded that a percutaneous approach should mainly be reserved for patients who cannot tolerate a laparoscopy or laparotomy. A multicenter retrospective study including 473 microwave ablations reported no superiority of the surgical approach over TA over OS or DFS, but surgical TA resulted in lower local recurrence (*p* = 0.014) without significantly increasing the complications rate [48]. Finally, a PSM study enrolling 168 patients investigated the effectiveness of the laparoscopic approach for TA, concluding that laparoscopic TA has the same efficacy as open surgical thermal ablations with less invasiveness [49]. However, laparoscopic TA is technically more challenging than percutaneous or open TA, owing to its different three-dimensional orientation, reduced freedom in needle angulation and orientation, and need to master intraoperative US. Giglio et al. reported a learning curve of 93 cases for laparoscopic TA to reduce the rate of incomplete ablations from 12.9 to 4.7% (0.027) [50].

The combination of RFA and LR has been applied in Asian countries in cases of more than three lesions to treat, for both HCC and CRLM, and has been shown to be safe and effective [51,52,53]. Zhou et al. published a study reporting the outcomes of combined LR-RFA for multiple HCC beyond the Milan criteria. In this paper, enrolling 469 consecutive patients, the 1-, 2-, and 3-year OS rates in the LR + RFA group were 81.8%, 68.7%, and 63.4%, vs. 59.3%, 36.1%, and 19.4% after TACE, after matching (*p* < 0.001). Subgroup analysis showed better outcomes after LR-RFA when including large tumors. No 30-day mortality was reported in the LR+RFA groups, vs. 1.22% after TACE.

Such encouraging results are in line with our experience as well. In particular, TA can be associated with laparoscopic liver resection (LLR), reaching the maximum benefit from the minimally invasive approach in cirrhotic patients [54]. Furthermore, the use of TA in combination with laparoscopic surgery can be helpful in the case of multiple lesions in different locations that are technically difficult to resect. The literature clearly shows that superficial or far-from-vessel lesions can be resected more easily with the laparoscopic approach, while deep and posterior ones can be complex to resect [55]. In these cases, TA can be used during laparoscopy to combine the technical advantages of both procedures if the size of the lesions to be ablated is <3 cm [56]. Further prospective studies on this type of combined approach are desirable, but both our experience and the sporadic evidence available allow for cautious optimism.

### 2.3. Role of Trans-Arterial Chemoembolization

TACE is considered the best therapeutic strategy for patients with intermediate-stage HCC [6]. The first trials supporting the role of TACE in this setting of patients compared TACE with best-supportive care for unresectable HCCs, showing longer survival rates for the TACE group [57,58]. However, there is heterogeneity in the intermediate BCLC-B stage with regard to liver function, tumor size, and tumor number. There is a strong need for a sub-classification, as testified by several attempts at creating a defined cutoff for subgroup division. Bolondi et al. firstly suggested to divide BCLC-B patients into four subgroups from B1 to B4 based on Child-Pugh stage, tumor size and number within or beyond up-to-seven criteria, and performance status, showing a different expected OS for the different subgroups, ranging from 31 months to only 10.9 months [59]. Similar differences in survival outcomes according to the different subgroups were reported by Ciria et al. [33]. According to Bolondi’s classification, TACE was recommended when liver function was preserved, while radio-embolization had to be preferred when tumor burden was beyond the up-to-seven criteria. However, there is no strong evidence about robust cutoffs, inducing the 2022 BCLC guidelines to suggest the adoption of an individualized patient profile to decide the best therapeutic option. In particular, the last recommendations stratify the BCLC-B stage into 3 subgroups of patients depending on liver function and tumor size and number. The first group comprises patients with well-defined HCC nodules that should undergo LT if they meet the extended criteria adopted in their country/institution [60]. The second subgroup includes patients not eligible for LT with a defined tumor burden and eligibility for TACE, which is the treatment of choice. To undergo TACE, HCC patients need to have well-preserved liver function; otherwise, there is a high risk of adverse events and poor outcomes [61]. If TACE is not feasible, the remaining BCLC-B patients should undergo systemic therapy. Several trials comparing TACE to systemic therapy and new immunotherapies for unresectable BCLC-B patients are still ongoing and may finally lead to a change in the management of such patients [62].

TACE refractoriness, defined as a non-responsivity to two TACE treatments, is another non-negligible issue. A study on 249 patients suggested a rate of refractoriness of 48.9% [63]. In these patients, a shift to sorafenib resulted in improved OS when compared to a further TACE attempt (25.4 months vs. 11.5 months, respectively, *p* = 0.003).

Drug-eluting bead TACE (DEB-TACE) has been proposed as a new and more effective technique. It entails the use of polymeric microspheres filled with chemotherapeutic drugs, which can slowly release the chemotherapeutic agent within the tumor area, potentially resulting in lower systemic toxicity and higher drug concentrations in the target tumor [64]. However, randomized controlled trials and retrospective studies failed to show a superiority of DEB-TACE over TACE in terms of long-term survival [65,66]. The only advantage shown by DEB-TACE was the lower post-procedural abdominal pain [65]. Transcatheter arterial chemo-infusion (TACI) is another variant of TACE that is not widely performed. It can release high concentrations of chemotherapeutic drugs in a highly selective manner, without performing the embolization. This could result in lower post-procedural pain and averse events, and could be proposed in patients with advanced disease and impaired liver function due to a lower risk of decompensation [67].

Downstaging is considered a viable option for selecting patients for LT, with the aim of reducing the tumor burden within transplant criteria [68]. TACE is the most widely performed downstaging method, with several sessions reported to be needed for effective downstaging [69]. Furthermore, there is wide heterogeneity among the available studies in this setting regarding surveillance protocols, embolic agents, chemotherapeutic agents, particle size, time between sessions, and indication for repeating therapy [70]. In addition, the limits for indicating the downstaging strategy are not clear and can vary among countries and institutions, depending on organ shortage and listing criteria [71]. Further studies with well-established designs is required to clarify the best timing and protocols, as well as the limits for the indications for a TACE-based downstaging treatment.

An interesting approach comes from Zhou et al. who proposed preoperative TACE for patients with intermediate-stage HCC, followed by LR [72]. This strategy resulted in higher OS (90.6% vs. 73.3% at 1 year, 61.7% vs. 43.5% at 3 years, and 52.9% vs. 33.8% at 5 years, respectively, *p* < 0.001) and DFS (54.6% vs. 39.4% at 1 year, *p* < 0.001, 41.4% vs. 29.4% at 3 years, *p <* 0.002, and 36.3% vs. 26.3% at 5 years, *p* = 0.008, respectively) than LR alone. This strategy is sometimes used in our institution after multidisciplinary team discussion in cases of multiple HCC and preserved liver function when LR is difficult, with good results. However, further studies are needed to obtain stronger evidence.

In conclusion, while TACE is a widely performed effective strategy for multiple HCC with preserved liver function, there are several issues that need to be clarified: the heterogeneity of the intermediate stage, risk factors, the management of TACE refractoriness (which may benefit from another treatment), and the exact limits within LR alone or combined with TACE that can achieve better survival outcomes. More robust evidence through a well-designed, randomized control study is definitely required.

### 2.4. The Role of Radiotherapy and Radioembolization

Some patients are not eligible for LT or LR due to impaired liver function, organ shortages, long waiting times, or late diagnosis [73]. In some reports, up to 20–25% of patients are not able to undergo any curative-intent treatment [74]. Radiotherapy could be an option for selected patients who are not eligible for other treatments.

Classically, radiotherapy for HCC was mainly indicated in the context of palliative care [75]. The main concern was radiation-induced liver disease (RILD), whose risk is higher when the whole organ is targeted. Finally, recent technological advances, such as stereotactic body radiation therapy (SBRT), allowed to target only the pathological areas, with a reduction of the radiation dose, decreasing the risk of RILD up to 5% [75]. Since its first description by Blomgren et al., SBRT has shown the advantage of precise tumor targeting with a step dose gradient, reducing the radiation to the surrounding normal parenchyma [76].

Some authors compared SBRT to TACE, as TACE is the treatment of choice for BCLC-B HCC patients. Epir et al. reported a better local control rate after SBRT than TACE (91% vs. 23%, *p* < 0.001), with similar survival outcomes (2-yr OS, 34.9% vs. 54.9%, *p* = 0.21) after matching 209 patients with less than three tumors [77]. SBRT and TACE were also compared in BCLC-B and -C patients, showing similar local control at 1-yr (82.9% vs. 84.8%, *p* = 0.8), as well as similar OS at 1-yr (52.9% vs. 53.1%, *p* = 0.61) [78]. These studies suggest SBRT could be an alternative approach to TACE in patients with BCLC-B HCC, with the possible advantage of avoiding the post-embolization syndrome. Ongoing studies are comparing TACE with SBRT (NCT02470533, NCT03338647). Furthermore, the addition of SBRT to TACE can achieve better treatment response, local control, and survival rates than SBRT alone [79]. Thus, further randomized studies are currently comparing TACE with TACE plus SBRT (NCT03895359 and NCT02794337).

Trans-arterial radioembolization (TARE), also known as selective internal radiation therapy (SIRT), is recognized as an alternative therapy for early and very early stage HCC not suitable for LR or TA [6]. In 2011, Salem et al. published a comparison between TARE and TACE in patients with unresectable HCC without extrahepatic metastasis [80]. Their results from the analysis on 463 patients showed a longer time-to-progression (TTP) after TARE (13.3 months) when compared to TACE (8.4 months, *p* = 0.046), but survival outcomes were comparable when focusing on patients with intermediate-stage disease (17.2 vs. 17.5 months, respectively). Post-procedural transaminase alteration was more frequent after TACE (*p <* 0.05). In 2016, the same group from Chicago Northwestern University published results from the first randomized controlled trial, showing a significant longer median TTP after TARE than TACE (>26 vs. 6.8 months, respectively; *p* = 0.0012). Results from comparisons of SBRT or TARE vs. TACE are shown in Table 2.

Furthermore, radiation lobectomy performed by TARE can control the local disease and induce a volumetric hypertrophy of the FLR, and this could be a very useful option in the case of an extensive resection in cirrhotic patients, such as in the case of multiple HCCs [86]. Finally, TARE has shown to be feasible and safe in patients with compromised liver function [81,87,88]. However, TARE is still recommended only in patients with BCLC stages 0 and A, within the Milan criteria, or with a giant solitary lesion. Results from larger perspective studies including patients with multiple HCC are still needed.

Finally, TACE still represents the therapy of choice for multiple HCC and compensated liver function, but, according to our experience and available literature, SBRT and TARE can play an important role in selected cases not eligible for LR [84,85]. A decisional algorithm is proposed in Figure 1. Further studies in this area are needed.

## 3. Minimally Invasive Approach for Multiple Hepatocellular Carcinoma

The role of MILS in multinodular HCCs is an important open issue. All the available literature comes from third-level referral centers. In 2012, our group published the first experience on LLR for multiple HCC [89]. Among 260 patients, the outcomes of LLR or LLR + TA were compared between patients with single tumors vs. multiple tumors. The two cohorts had comparable clinical and pathologic characteristics, except for a higher rate of previous TACE in the multiple HCC group. No significative differences were found in the rate of intraoperative transfusion, length of postoperative hospital stay, mean operative time, or postoperative complications. Obviously, laparoscopic TA was more commonly used for multiple HCCs. No significative difference in OS was found after a median follow-up of 33.7 months, but DFS was lower in the group with a single lesion.

A further PSM study enrolling 150 patients reported similar complication rates, as well as OS (*p* = 0.502) and DFS (*p* = 0.887) between LLR and open liver resection (OLR) for multinodular HCC, with a significantly shorter length of hospital stay after LLR (median, 7 vs. 8 days, respectively, *p* = 0.014) [90].

In our experience, LLR for multinodular HCC is safe and feasible. However, some precautions are essential to reaching adequate oncologic outcomes, such as a high expertise in ultrasonography-guided parenchymal dissection with intraoperative ICG-guided fluorescence that can further help detect HCC nodules and guide difficult parenchymal dissection, while 3D-high definition scopes could represent an additional supportive visual tool [91,92]. Further technological research is supposed to help surgeons in this scenario, such as the application of 3-D preoperative modeling and virtual realities, which could also be beneficial in this context [93].

Finally, an interesting recent PSM study compared LLR and OLR for BCLC-B patients with resectable multiple HCC, showing better perioperative outcomes for the minimally invasive approach in selected patients [94]. In particular, median estimated blood loss (200 vs. 350 mL, *p* = 0.005) was lower after LLR, with similar complication rates (*p* = 0.035), OS (*p* = 0.827), and DFS (*p* = 0.694). The mean operation time was shorter after OLR (237.5 vs. 210 min, *p* = 0.024). Interestingly, the rate of postoperative ascites was 0% after LLR in the BCLB-B patients vs. 11.3% after OLR (*p* = 0.06).

In conclusion, in high volume referral centers, LLR (±TA) should be considered in cases of multinodular HCCs suitable for LR, because of the potential advantages over OLR, particularly in the subset of Child-B cirrhotic patients [95]. A personalized strategy, with the combination of LLR and TA, should always be proposed to overcome some technical issues about deep and posterior lesions while maintaining the advantages of a minimally invasive approach [96]. More robust studies are needed to support clinical practice.

### The Role of Robotic Liver Resection

Although robotic surgery is rapidly expanding in minimally invasive liver surgery, there are still concerns about long-term outcomes, especially for complex procedures such as multiple resections [97]. In such cases, the robotic platform can provide useful tools for the visualization of the multiple lesions, such as high-definition ICG-fluorescence thenks to the *firefly* system, as well as the 3D navigation integration *tylepro* program [98].

There are still no studies in the literature focusing specifically on the robotic approach for multiple liver tumors, including HCC. However, the most recent series include resection of multiple HCC in their population and show very encouraging results [99]. Indeed, robotic liver resection (RLR) can ideally overcome some limitations of LLR, such as the lack of flexibility of the operating instruments, due to the ability to articulate the instruments because of the 360° of freedom for the surgeon’s wrist and a magnified high-definition vision, as well as to considerable ergonomic advantages [100,101]. Recently, a meta-analysis including 487 RLR concluded for lower bleeding rates after RLR at the expense of a longer operation time [102].

Therefore, some advantages could be cautiously hypothesized for multiple HCC, but more evidence is required. Furthermore, the expensive costs and the organizational and logistic aspects are still important drawbacks for the further expansion of the indications of RLR.

## 4. Conclusions

In conclusion, the latest evidence indicated that LR could provide better survival outcomes in selected patients with multiple HCC staged as BCLC-B or -C when compared to TACE, as already acknowledged by the Asian Pacific Association for the Study of the Liver (APASL) and the latest recommendations of the European Society for Medical Oncology (ESMO) [12,103].

In this scenario, LLR has been reported to have encouraging results and can be associated with laparoscopic TA to maximize the benefits of a minimally invasive approach while overcoming some technically challenging situations in the case of multiple HCCs not eligible for surgery. SIRT and SRBT can play an important role, together with the consolidated TACE. Furthermore, larger prospective studies on the treatment of multiple non-metastatic HCC should be conducted.

## Figures and Tables

**Table 2:** Studies comparing SBRT or TARE vs. TACE in BCLC-B patients. OS: overall survival; SBRT: stereotactic body radiation therapy; TARE: trans-arterial radioembolization; TACE: trans-arterial chemoembolization; HCC: hepatocellular carcinoma; BCLC: Barcelona clinic liver cancer.

Year	Authors	Study Design	Compared Groups	Sample Size	Inclusion Criteria	1-yr OS (%)	3-yr OS (%)	Median OS (Months)	*p*-Value
2011	Salem et al. [80]	Retrospective	TARE vs. TACE	123 vs. 122	Non-metastatic, inoperable HCC with preserved liver function without portal vein thrombosis			20.5 vs. 17.4	0.23
2013	Moreno-Luna et al. [81]	Retrospective frequency- matched	TARE vs. TACE	61 vs. 55	Inoperable, non-metastatic HCC		21 vs. 16		0.47
2015	Pitton et al. [66]	Randomized controlled trial	TARE vs. DEB-TACE	12 vs. 12	Non-metastatic, inoperable BCLC-B patients			19.7 vs. 26.6	0.92
2015	Kolligs et al. [82]	Randomized controlled trial	TARE vs. TACE	13 vs. 15	Non-metastatic HCCs, Child-Pugh ≤B7, ≤5 liver lesions, ≤20 cm total maximum diameter	46.2 vs. 66.7			n.a.
2016	Salem et al. [83]	Randomized controlled trial	TARE vs. TACE	24 vs. 21	Inoperable, non-metastatic BCLC-A/B patients without portal vein thrombosis			18.6 vs. 17.7	0.99
2018	Sapir et al. [77]	Retrospective propensity score—matched	SBRT vs. TACE	125 vs. 84	Non-metastatic HCC	74.1 vs. 75.3			0.21
2018	Bettinger et al. [78]	Retrospective propensity score -matched	SBRT vs. TACE	35 vs. 367	Non-metastatic HCC	31.4 vs. 54.2			0.49
2019	Shen et al. [84]	Retrospective propensity score- matched	SBRT vs. TACE	46 vs. 142	Single or multiple medium sized HCCs (3–8 cm)		55 vs. 13		0.001
2020	Su et al. [85]	Retrospective propensity score- matched	SBRT vs. TACE	167 vs. 159	Inoperable BCLC-A HCCs	85.7 vs. 83.6	65.1 vs. 61		0.29

## Data Availability

Not applicable.
